# Supplementary material for: Content-rich biological network constructed by mining PubMed abstracts
Source: BMC Bioinformatics. 2004 Oct 8;5:147. doi: 10.1186/1471-2105-5-147 (PMC528731; doi:10.1186/1471-2105-5-147)
Supplement: Additional File 2 — The original results of the above study (non-essential files are deleted to keep the file size under the limit set by BMC bioinformatics). [file 1471-2105-5-147-S2.bz2 › chilibotAdditionalFile2/dip05/451ID10477718E10462/html/CCL7_CCR5.html]

 


 **CCL7** and **CCR5** 
  
Found 35 abstracts in PubMed, retrieved 05.  
 

 What does Google say? 
 PDF only 
| .edu only 

---

**Interactive relationship** (e.g. stimulation, inhibition, etc)

**Non-interactive relationship** (e.g. studied together, co-existance, homology, etc.)

- In parallel with down regulated SP and  **CCR5**  mRNA expression, cDNA array assays indicated that CP 9 45 treatment also inhibited IL 8 gene expression, while enhancing expression of fractalkine and monocyte chemotactic protein 3  [ **CCL7** ]  MCP 3  [ **CCL7** ] .  Ref: 12098517 J Neuroimmunol, 2002
- MIP 1alpha, MIP 1beta, RANTES, MCP 2 and MCP 4 were able to stimulate increases in intracellular calcium ions via activation of  **CCR5**  whereas MCP 3  [ **CCL7** ]  was without effect.  Ref: 11861332 Br J Pharmacol, 2002
- We investigated the effects of a number of naturally occurring chemokines MIP 1alpha, MIP 1beta, RANTES, MCP 2, MCP 3  [ **CCL7** ] , MCP 4 on different processes linked to the chemokine receptor  **CCR5**  in recombinant CHO cells expressing the receptor at different levels.  Ref: 11861332 Br J Pharmacol, 2002
- A number of inflammatory CC chemokines, including MIP 1 alpha, MIP 1 beta, RANTES, MCP 2, and HCC 1 9 74 act as  **CCR5**  agonists, while MCP 3  [ **CCL7** ]  is a natural antagonist of the receptor.  Ref: 12402506 Receptors Channels, 2002
